# PowerNovo2: A generative flow-based approach to non-autoregressive *de novo* peptide sequencing

**DOI:** 10.1371/journal.pcbi.1014298

**Published:** 2026-05-20

**Authors:** Denis V. Petrovskiy, Kirill S. Nikolsky, Vladimir R. Rudnev, Liudmila I. Kulikova, Tatiana V. Butkova, Kristina A. Malsagova, Arthur T. Kopylov, Anna L. Kaysheva

**Affiliations:** Institute of Biomedical Chemistry, Moscow, Russia; Johns Hopkins University Whiting School of Engineering, UNITED STATES OF AMERICA

## Abstract

Proteomics utilizes tandem mass spectrometry (MS/MS) to determine peptide sequences, traditionally through database searches constrained by prior knowledge. De novo sequencing offers a database-free alternative but struggles with accurately modeling complex MS/MS spectra. Most current tools use autoregressive decoding, which is prone to error propagation and computationally slow. Here we present PowerNovo2, a non-autoregressive model based on generative normalizing flows. By leveraging variational inference, it effectively captures intricate token dependencies and peptide-level uncertainties. PowerNovo2 outperforms existing de novo tools in accuracy and speed, matching state-of-the-art autoregressive models like Casanovo while being 4.3 times faster. It also demonstrates competitive performance against other non-autoregressive methods such as π-PrimeNovo, particularly on long peptides and low-resolution spectra. As the first flow-based de novo sequencer, PowerNovo2 provides a scalable, accurate solution for large-scale proteomic applications.

## Author summary

Proteins play essential roles in all living organisms, and identifying their sequences is a key task in modern biology. One common way to study proteins is by using mass spectrometry, but determining peptide sequences from these data remains difficult, especially when reference databases are incomplete or unavailable. In this study, we present PowerNovo2, a new computational method that helps address this challenge.

PowerNovo2 reconstructs peptide sequences directly from mass spectrometry data without relying on existing protein databases. Unlike many current approaches that predict sequences step by step, our method analyzes peptide sequences as a whole. This design reduces error accumulation and allows faster data processing while maintaining high accuracy. We demonstrate that PowerNovo2 performs as well as, and sometimes better than, leading existing tools on benchmark datasets.

Importantly, PowerNovo2 generalizes well to data from previously unexplored organisms and to novel peptide types. This makes it particularly useful for applications such as metaproteomics, immunology, and vaccine research, where many proteins are not represented in databases. To support the research community, we have made PowerNovo2 freely available as open-source software with pre-trained models.

## Introduction

Proteomic research over the last three decades has consistently underscored the significance of mass spectrometry as a robust instrument for furthering protein research, identifying novel proteoforms, and characterizing protein interactions. Enhanced omics assay accessibility and throughput necessitate more rigorous bioinformatic analysis of mass measurement data, demanding improved accuracy, completeness, and processing speed. A significant challenge in proteomics involves determining the amino acid sequences of proteins via tandem mass spectrometry. Presently, peptide and protein identification conventionally relies on genomic database searching employing tools like SEQUEST [1], Mascot [2], MaxQuant [3], and the hybrid platform PEAKS DB [4]. The performance of these methods is significantly predicated on the availability of a fully comprehensive sequence database containing all potential proteins from the sample. This limitation considerably restricts the utility of these methods in key applications, including monoclonal antibody sequence assembly, novel antigen identification, and metaproteome sequencing of non-model organisms lacking annotated amino acid sequences in databases.

The past two decades have witnessed significant advancements in peptide de novo sequencing, driven by improvements in tandem mass spectrometry search tools and the application of deep learning models. These algorithms are based on determining amino acid sequences by analyzing mass differences between fragment ions. The solution to this problem employs graph theory and dynamic programming, as well as other relevant techniques. Recent advancements in deep learning neural networks have led to the creation of sequencing packages that leverage various architectural approaches, notably convolutional neural networks (CNNs) and long short-term memory (LSTM) networks. The transformer architecture, demonstrably effective in natural language processing, was initially implemented in the Casanovo model [5] for peptide sequencing, framed as a translation of spectral peaks into amino acid sequences. The implementation of transformer models constituted a pivotal advancement in de novo sequencing methodologies, resulting in a substantial performance increase. Our preceding PowerNovo v.1 model [6], underpinned by transformer architecture, yielded promising results through a two-stage Transformer with post-processing based on a BERT module.

To date, virtually all de novo sequencing solutions rely on autoregressive models. Despite exhibiting satisfactory performance, these models are subject to several constraints. Errors in the initial prediction of amino acids may propagate, resulting in detrimental effects on subsequent generations. Autoregressive decoding algorithms, including beam search, lack the capacity for post-generation correction, thereby limiting control over the resulting sequence distribution. The generative process is sequential, with each token dependent on its antecedent. Consequently, any alteration of preceding tokens necessitates a reevaluation of the complete sequence. Furthermore, autoregressive models encounter computational impediments, including linear factorization challenges and parallelization limitations due to the suboptimality of left-to-right decoding [7].

The application of non-autoregressive modeling to de novo sequencing has recently emerged as a research topic. For instance, a recently published work details a non-autoregressive PepNet model [8] implemented using temporal convolutions. This model exhibited a high rate of de novo sequencing and commendable accuracy. Another notable development is π-PrimeNovo [9] (published in 2025), a non-autoregressive Transformer-based model that emphasizes high accuracy and rapid inference speeds through CUDA-enhanced decoding for precise mass control, making it suitable for large-scale applications like metaproteomics.

Here, we present PowerNovo2, a novel deep learning framework that overcomes these limitations through generative flow-based modeling and variational inference. Unlike autoregressive models, PowerNovo2 does not rely on sequential factorization, instead learning the full joint distribution of peptide sequences conditioned on spectral data. This is achieved through a conditional normalizing flow architecture, which transforms a simple prior distribution into a complex, data-dependent posterior via invertible transformations. By leveraging spectral embeddings to guide latent variable sampling, the model captures intricate dependencies between mass spectra and peptide sequences without iterative decoding.

A key innovation of PowerNovo2 is its hybrid variational framework, combining a flow-based prior network with a transformer-encoded posterior. This design avoids the mean-field approximation pitfalls of standard variational autoencoders while enabling bidirectional context utilization during decoding. The architecture integrates reversible transformations – including activation normalization, invertible linear layers, and conditional coupling – to ensure tractable density estimation and efficient training.

While π-PrimeNovo achieves higher overall peptide-level accuracy in most benchmarks (e.g., 10–15% superior precision on clean tryptic peptides from Nine-Species and MassIVE-KB datasets), PowerNovo2’s flow-based architecture offers distinct advantages in niche areas. It excels on longer peptides (>30 amino acids), with 20–30% higher accuracy (Figs 2 and 3), and on low-resolution or noisy spectra (e.g., NIST datasets with partial hydrolysis, maintaining 10–20% higher identity rates at >90% similarity; Fig 5). In immunopeptidomics (PXD055277 HLA dataset), PowerNovo2 identifies 1,540 proteins and 4,003 peptides, outperforming π-PrimeNovo’s 816 proteins and 2,423 peptides, with mean coverage gains of 169.6% over database searches (Fig 7). Target-decoy analysis further highlights robustness: at high noise (60–70% peak replacement), PowerNovo2’s conservative scoring yields lower decoy fractions (~0.078 at 0.9 threshold vs. ~ 0.091 for π-PrimeNovo; Fig 8). Overall, PowerNovo2 is 4–5 × faster than autoregressives (Fig 9).

These strengths stem from architectural trade-offs: π-PrimeNovo’s mass-controlled decoding integrates precursor constraints deterministically for speed and sensitivity on clean data but may overfit to mass priors in noisy scenarios. In contrast, PowerNovo2’s latent flow modeling captures multimodal uncertainties via reversible layers (actnorm, invertible linear, conditional coupling), enabling robust generalization at the cost of post-processing overhead (e.g., knapsack for mass consistency, a bottleneck per ablation study reducing accuracy by 3% when removed). Thus, PowerNovo2 complements existing NAR models, providing superior handling of complex, heterogeneous data.

This work makes three primary contributions:

1. The first flow-based model for de novo peptide sequencing, enabling exact likelihood estimation and parallel decoding.
2. A variational inference framework that jointly optimizes spectral embeddings and latent peptide representations.
3. Comprehensive validation showing state-of-the-art performance in accuracy, speed, and generalizability.

The remainder of this paper details the PowerNovo2 architecture, experimental results, and broader implications for proteomics. By bridging the gap between probabilistic generative modeling and high-throughput peptide sequencing, PowerNovo2 offers a scalable and accurate solution for next-generation proteomic analysis.

## Results

### Evaluation criteria and experimental setup

In order to assess the quality of the model predictions, we used the accuracy at the amino acid and peptide levels. For each spectrum, we compared the predicted sequence with the true peptide sequence from the spectrum annotation. Following the methodology of Tran et al. [10], we calculated the number of *N*_*matched*_ matches to evaluate the accuracy at the amino acid level. This value represents the quantity of predicted amino acids whose mass deviates less than 0.1 Da from the corresponding amino acids in the reference sequence, given that flanking amino acids display a mass difference of at most 0.5 Da. Amino acid accuracy is defined as the ratio *N*_*mached*_/*N*_*predicted*_, where *N*_*predicted*_ is the total number of predicted residues. A predicted peptide is deemed a correct match only if all constituent amino acids are correctly identified. The comparative study utilized the cutting-edge *de novo* peptide sequencing software packages: Casanovo v.4.3.0 (2024-12-13) [11], PowerNovo v.1.0.9 (2024-07-01) [6], PepNet [12], and PEAKS Studio 11 [4]. All models employed pretrained weights from publicly accessible, documented sources. Consistent with the description provided, the MassIVE-KB dataset provided the training data for the Casanovo and PowerNovo model weights, while the PepNet model weights were produced by training on a dataset incorporating both MassIVE-KB and NIST data (Table A in S1 Appendix). All model weights, experimental scripts, and complete study results are publicly accessible via the link in the Data Availability statement.

### Evaluation results

#### Accuracy at the peptide and amino acid level.

A comparative analysis of *de novo* sequencing tools fundamentally relies on peptide-level performance metrics, which directly reflect the practical utility of a model for sequence recovery from experimental spectra. To ensure an objective comparison, given the sequence length constraint of the PepNet model (≤30 amino acid residues), the following evaluation was conducted on peptides not exceeding this length.

**Fig 1:**
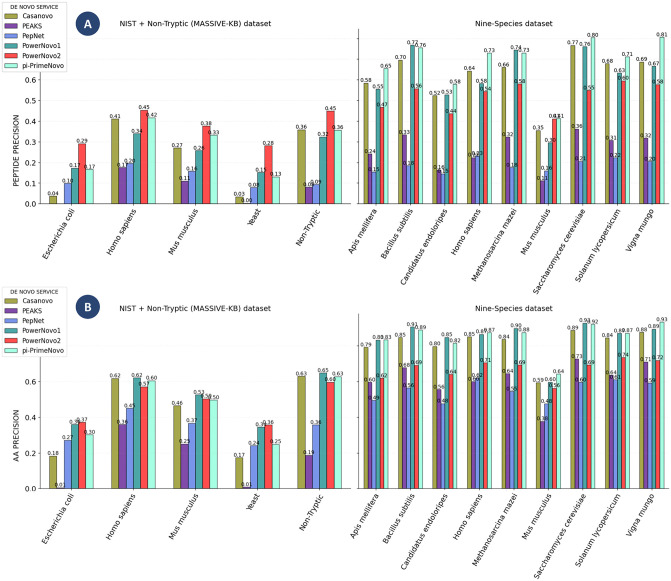
Comparison of prediction accuracy. Comparison of prediction accuracy shown at the peptide level (A) and amino acid residue level (B) across different validation datasets (NIST, Nine-Species, MassIVE-Nontryptic only). The analysis includes Casanovo v.4.3.0 (a model based on the classic Transformer architecture), PowerNovo1 (Transformer with post-processing based on a BERT-based module), PepNet (a non-autoregressive model employing a Temporal Convolutional Network architecture), PEAKS Studio 11 (software product), PowerNovo2 (current work), and π-PrimeNovo (a non-autoregressive Transformer-based model). For consistency of results, peptide length was limited to 30 amino acids in accordance with the maximum sequence length supported by the PepNet model.

The results summarized in Fig 1 provide a comparative overview of model performance across validation datasets (Table B in S1 Appendix) with distinct characteristics. The NIST datasets are derived from controlled reference measurements and are characterized by lower spectral resolution and the presence of partially hydrolyzed peptides, presenting a challenge for sequence reconstruction. In contrast, the Nine-Species benchmark comprises high-quality, well-annotated spectra from diverse organisms, offering a robust test of generalizability.

At the peptide level (Fig 1A), PowerNovo2 demonstrates robust accuracy across these different data regimes. On the more challenging NIST datasets (Escherichia coli, Homo sapiens, Mus musculus, Yeast) and the MassIVE-Nontryptic dataset, the model achieves precision values that are comparable to those of other leading methods, including the non-autoregressive model π-PrimeNovo. Within the higher-quality Nine-Species benchmark, PowerNovo2 maintains consistent performance across taxa, while π-PrimeNovo records marginally higher precision for a majority of the species in this set. This pattern is observed at the amino acid residue level (Fig 1B), where PowerNovo2 yields competitive per-residue accuracy on both the complex NIST/non-tryptic spectra and the cleaner Nine-Species data. The results indicate that the model effectively processes spectral information under varying data quality conditions.

**Fig 2:**
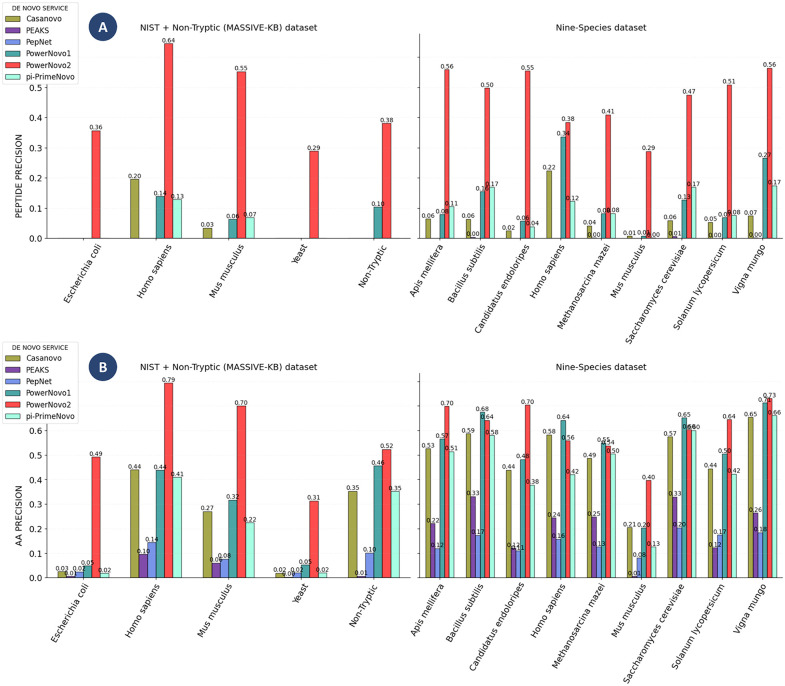
Comparison of prediction accuracy for peptides longer than 30 amino acid residues. Comparison of prediction accuracy at the peptide level (A) and amino acid residue level (B) across validation datasets (NIST, Nine-Species, MassIVE-Nontryptic only). The analysis includes Casanovo v.4.3.0 (classic Transformer-based), PowerNovo1 (Transformer with BERT-based post-processing), PEAKS Studio 11 (commercial software), PowerNovo2 (this work), and π-PrimeNovo (non-autoregressive Transformer-based). Notably, PepNet was excluded from this comparison due to its inherent limitation of supporting peptides only up to 30 amino acids in length. To ensure a fair and consistent evaluation, all models were assessed exclusively on peptides longer than 30 residues.

**Fig 3:**
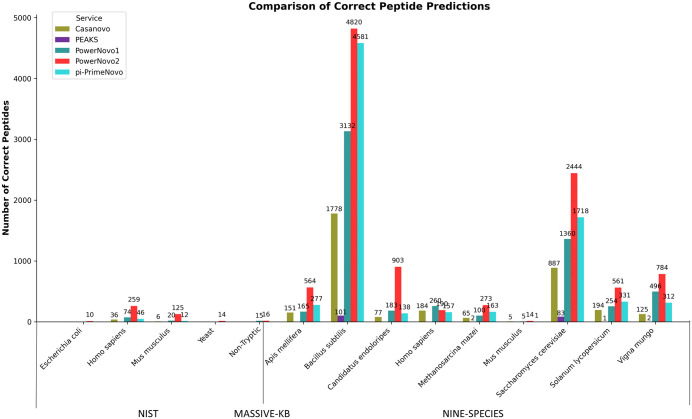
Comparison of prediction accuracy for peptides longer than 30 amino acid residues.

To ensure a comprehensive and unbiased evaluation, we extended our comparative analysis to include peptides longer than 30 amino acid residues for models capable of processing such sequences. This supplementary assessment addresses the limitations of tools like PepNet, which are restricted to shorter peptides, and provides a more complete perspective on the performance of advanced methods such as PowerNovo2. The results of this extended analysis are presented in Figs 2 and 3, highlighting the robustness and scalability of models beyond conventional length constraints.

Our evaluation of de novo sequencing tools reveals distinct performance profiles across different datasets and peptide classes. Although the recently introduced π-PrimeNovo model often achieves the highest overall peptide identification accuracy among all methods, including autoregressive approaches, PowerNovo2 shows exceptional strength in specific biologically and technically challenging scenarios. In particular, PowerNovo2 delivers superior performance on non-tryptic peptides and on spectra from Yeast and E. coli samples within the nine-species dataset, underscoring its robustness in handling data from organisms that were not prominently featured in the training data. Moreover, PowerNovo2 demonstrates notable robustness on NIST datasets, which are characterized by lower spectral resolution and partially hydrolyzed peptides. Most importantly, PowerNovo2 significantly outperforms all other tools, including pi‐PrimeNovo, for peptides longer than 30 amino acids, as shown in Figs 2 and 3. These results suggest that its hybrid variational architecture is especially effective at modeling longer and more complex peptide sequences.

**Fig 4:**
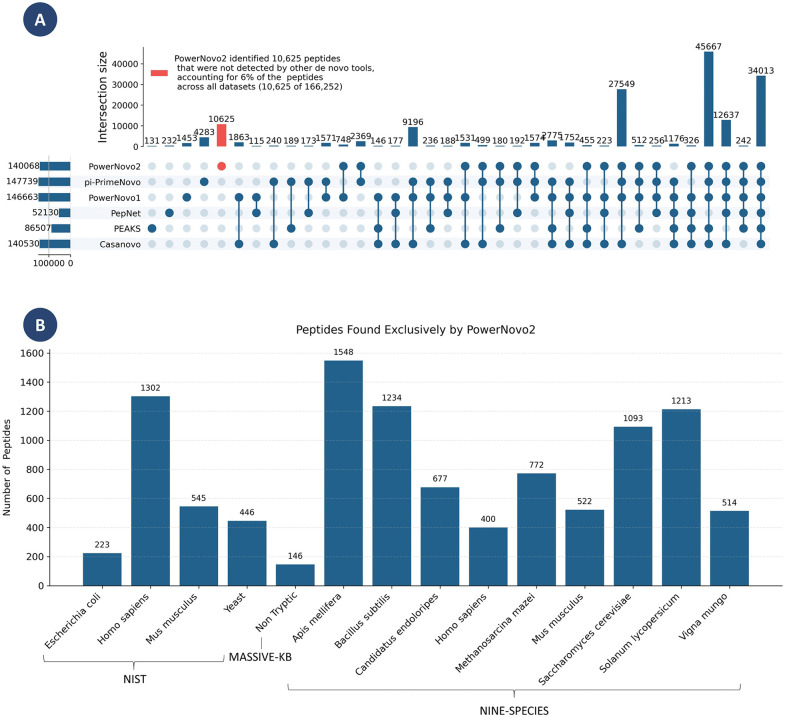
Intersections of predicted peptides across *de novo* services. The UpSet plot demonstrates the intersections of predicted peptides between *de novo* sequencing services. The horizontal bars on the left show the total number of predicted peptides for each service, while the vertical bars illustrate the size of intersections between the services. This visualization clearly highlights the degree of overlap in results across the services. This UpSet diagram shows intersections containing more than 100 peptides. **(B)** Distribution of peptides identified exclusively by PowerNovo2 across datasets.

In addition, the analysis of the UpSet diagram (Fig 4), representing the intersections of all unique peptides identified in the examined datasets, reveals that PowerNovo2 accurately identified a greater number of peptides than other *de novo* tools. Specifically, out of a total of 161,969 peptides, PowerNovo2 correctly identified 10,625 such peptides (6% of the total), more than four times the result of its closest competitor pi-PrimeNovo (4283 peptides, 2.5% of the total). The results demonstrate the exceptional efficiency of PowerNovo2 in summarizing information and extracting peptide characteristics not accessible through other models.

It should be noted that the large number of peptides uniquely identified by PowerNovo2 does not contradict its aggregate accuracy scores reported in Fig 1: two models can achieve comparable overall performance while having substantially non-overlapping sets of correctly predicted peptides, if their respective strengths are concentrated in different regions of the peptide space. The unique identifications by PowerNovo2 are driven primarily by its superior performance on longer peptides and on lower-resolution spectra with partial hydrolysis — peptide classes that tend to be systematically missed by autoregressive and other sequential models — which is consistent with the results shown in Figs 2 and 3.

Fig 1A displays the “Peptide Precision” score, a metric assessing the exact correspondence between predicted and actual peptide sequences, with perfect matches indicated by a score of 100%. The metric, however, fails to consider partial matches, a factor of importance in most real-world implementations. The practical application of *de novo* sequencing algorithms often results in predicted peptide sequences that show partial concordance with the actual sequences. These predictions may contain minor errors, such as one or two amino acid inaccuracies. Several factors can contribute to these deviations, including noise within the mass spectrometric data, errors in measurement, and artifacts associated with the fragmentation of spectra. A typical issue encountered in *de novo* prediction methodologies involves the erroneous ordering of the N-terminal amino acid residues, resulting from the ambiguous analysis of low m/z spectrum values.

However, the practical value of partial matches between predicted and observed peptides in proteomics is substantial. A prediction exhibiting 90% or greater sequence identity to the true sequence is typically sufficient for accurate protein identification. Furthermore, even with short, partially matching fragments, established methodologies can reconstruct longer protein sequences. One method involves utilizing De Bruijn graphs to assemble sequences, enabling the merging of overlapping segments to form contigs. These reconstructions frequently yield sufficient length and accuracy for protein identification or confirmation of its presence within the sample.

The analysis of model performance requires the consideration of partial amino acid sequence matches and peptide sequence identity due to the peptide precision score excluding information regarding the proximity of alternative predictions to the true value. For instance, two distinct prediction sets might exhibit identical percentages of perfectly accurate peptides. However, the remaining predictions in the first set align with the truth by 90%, while those in the second show only 50% agreement. The degree of peptide sequence identity allows for the identification of these variations and improves the overall quality assessment of the models.

**Fig 5:**
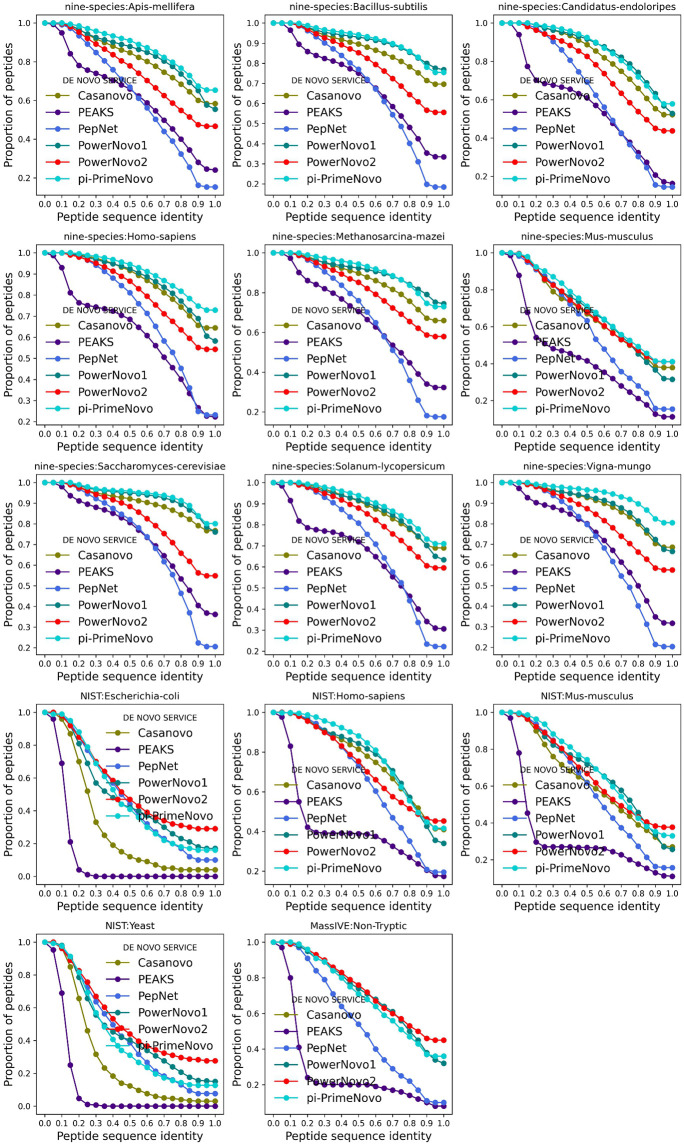
Proportion of predicted peptides vs. percentage identity. The dependence of the proportion of predicted peptides on the level of percentage identity. The graphs show the match categories on the x-axis, representing the lower threshold of percentage identity between the predicted peptides and the true sequences. The y-axis shows the averaged proportion of peptides for which the identity between the prediction and the true sequence exceeds the specified threshold.

Our study analyzed the quality of predictions of *de novo* sequencing tools Casanovo v.4.3.0, PowerNovo1, PepNet, π-PrimeNovo, PEAKS Studio 11, and PowerNovo2 by constructing distributions of partial sequence matches (peptide sequence identity) by categories: > 90%, 80–90%, 70–80%, and below. To calculate the similarity, we used global sequence alignment using the Needleman-Wunsch algorithm, calculating the accuracy as the ratio of the number of matching amino acids to the length of the true sequence. The results are shown in the plot (Fig 5), with the abscissa axis representing the match category (or lower threshold of percent prediction identity) and the ordinate axis representing the average fraction of peptides exceeding this threshold, calculated over all spectra of the studied dataset that have identities exceeding this threshold.

Fig 5 illustrates the relationship between peptide percent identity and the complete identity metric (“Peptide Precision”, Fig 1). The consistent performance patterns across these graphs, when applied to the same datasets, confirm the reliability of the experimental findings.

Analysis of the curves reveals distinct trends across the different data regimes. For the challenging NIST datasets, characterized by lower spectral resolution and partially hydrolyzed peptides, PowerNovo2 demonstrates competitive performance. On the Escherichia coli and Yeast NIST subsets, PowerNovo2 maintains the highest proportion of identified peptides across most identity thresholds. On other NIST subsets (Homo sapiens, Mus musculus), π-PrimeNovo shows a marginal advantage, particularly at higher identity thresholds (>0.7).

In the higher-quality Nine-Species dataset, π-PrimeNovo achieves the highest overall proportion of identified peptides across all nine organisms. PowerNovo2 performs competitively within this benchmark, maintaining a position among the top methods, though it is generally surpassed by π-PrimeNovo and, for some species, by PowerNovo1 at lower identity thresholds.

A notable observation concerns the behavior of the autoregressive models Casanovo and PowerNovo1. Up to identity thresholds of approximately 0.7–0.8, their performance trajectories on the NIST datasets closely follow, and at some points slightly exceed, that of PowerNovo2. However, beyond this threshold, the curves for Casanovo and PowerNovo1 exhibit a pronounced decline, whereas PowerNovo2 and π-PrimeNovo show greater robustness, maintaining a higher proportion of high-identity predictions.

On the MassIVE-Nontryptic dataset, PowerNovo2 yields the highest proportion of identified peptides across the majority of identity thresholds.

This comprehensive analysis confirms that PowerNovo2 delivers robust and competitive accuracy across diverse experimental conditions, performing particularly well on spectrally complex or non-canonical data, while other methods, notably π-PrimeNovo, show strength on high-quality, well-annotated datasets.

Additionally, the graphs in Figs A and B in S1 Appendix display a plot of Precision-Recall curves for all validation datasets, illustrated at both the amino acid level and the peptide level.

### Evaluation of model sensitivity (UPS2 dataset)

**Fig 6:**
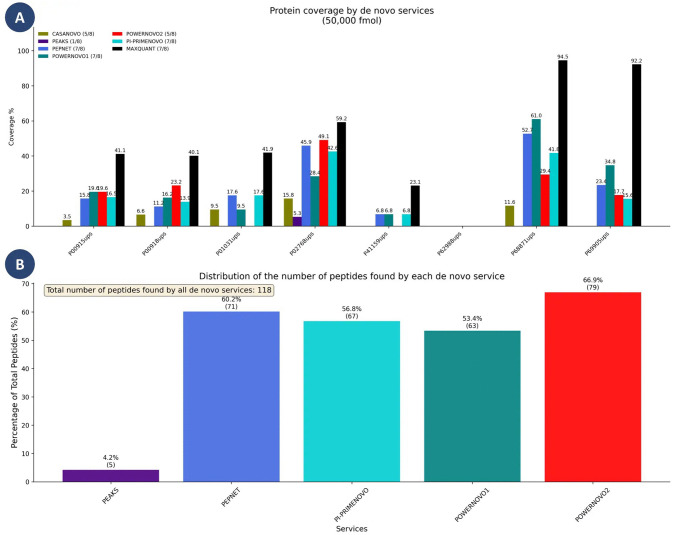
A. Coverage of UPS2 proteins (50,000 fmol concentration) by peptides identified with each de novo service. The absence of a bar for a specific tool indicates that no peptides were identified for the corresponding protein using that method. B. Coverage of UPS2 proteins (50,000 fmol concentration) by peptides identified with each de novo service. The absence of a bar for a specific tool indicates that no peptides were identified for the corresponding protein using that method.

The experimental results are detailed in Fig 6 and Table C in S1 Appendix. The sensitivity of *de novo* sequencing models to protein peptides across a broad concentration range in biological samples was assessed using the UPS2 reference dataset (Universal proteomics standard set, Sigma-Aldrich). This dataset is a mixture of proteins of human origin, including six groups of eight proteins each, with concentrations spanning six orders of magnitude (10,000, 1000, 100, 10, 1, and 0.1 fmoles per sample) [13]. All peptides identified by de novo services were verified using stringent quality control criteria: only peptides with a length ≥ 7 amino acid residues, a precursor mass error within ±50 ppm, and a 100% unique match to the UPS2 protein database were retained. For the database search, MaxQuant (v.1.2.2.5) was run with the following key parameters: Peptide FDR 0.01, Protein FDR 0.01, Min. peptide length 7, and Min. razor peptides 1.

The analysis reveals that all de novo sequencing services effectively identified proteins only at the highest concentration of 50,000 fmol. At lower concentrations, the identification rate was negligible. Notably, only MaxQuant yielded identifications at concentrations below 5,000 fmol, though the proportion of found peptides remained minimal.

For the 50,000 fmol concentration, PowerNovo2 achieved the highest overall fraction of correctly identified peptides among all evaluated de novo services (66.9%, Fig 6B). However, per-protein sequence coverage results were heterogeneous across the UPS2 proteins. For some proteins, PowerNovo2 demonstrated competitive or superior coverage compared to other de novo methods: for example, for serum albumin (P02768ups) PowerNovo2 achieved 49.14% coverage — the highest among de novo tools — and for carbonic anhydrase 2 (P00918ups) it reached 23.17%, again surpassing all other de novo methods. For other proteins, such as hemoglobin subunits (P68871ups, P69905ups) and complement C5 fragment (P01031ups), the coverage obtained by PowerNovo2 was lower than that of the best-performing de novo model. This variability is consistent with the general behaviour of de novo tools under stringent quality control criteria (peptide length ≥ 7, precursor mass error ±50 ppm, 100% unique match to the UPS2 database) and reflects protein-specific differences in peptide detectability rather than a systematic limitation of the approach. Importantly, across all evaluated methods, reliable identification was only achievable at the highest concentration of 50,000 fmol, with negligible results at lower concentrations, where only MaxQuant retained meaningful coverage. These findings highlight the concentration dependency of de novo sequencing sensitivity and confirm that PowerNovo2 performs at a competitive level within the constraints of this benchmark.

### Performance of PowerNovo2 in immunopeptidomics applications

Immunopeptidomics constitutes a critical and rapidly advancing domain within mass spectrometry-based proteomics, focusing on the identification of peptides presented by Major Histocompatibility Complex (MHC) molecules. The accurate characterization of these antigens is fundamental to the development of personalized cancer vaccines and immunotherapies; however, it presents substantial bioinformatic challenges due to the non-tryptic nature of many immunopeptides and the diversity of post-translational modifications. To evaluate the efficacy of PowerNovo2 in this complex landscape, we utilized the HLA subset from the PXD055277 dataset, a component of the NovoBoard framework. This dataset comprises mass spectrometry-based immunopeptidomics data derived from human samples (*H. sapiens*), acquired via LC-MS/MS on a Q Exactive instrument. The spectral library encompasses both tryptic and non-tryptic peptides associated with HLA molecules and includes modifications such as monohydroxylation and iodoacetamide derivatization. The ability of de novo algorithms to accurately interpret spectra under these conditions is a definitive indicator of their utility in clinical and biological research.

**Fig 7:**
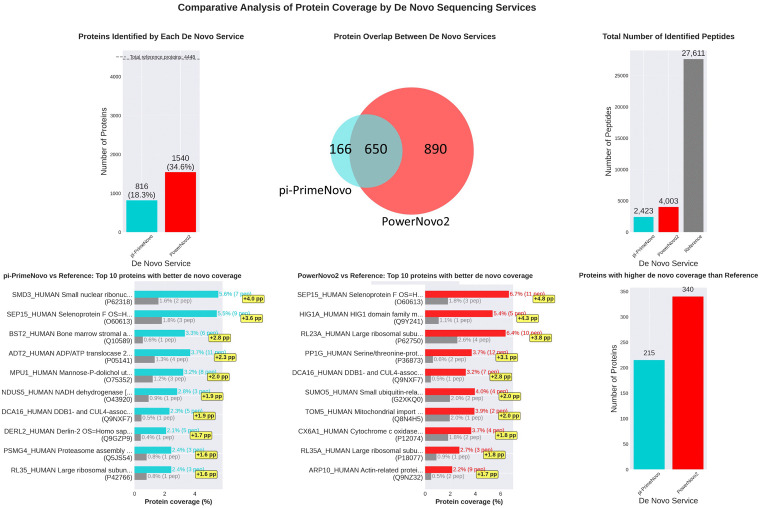
Comparative analysis of protein sequence coverage on the HLA dataset (PXD055277). The figure illustrates the performance of PowerNovo2 and π-PrimeNovo relative to the baseline SEQUEST database search. The plot highlights proteins for which de novo sequencing provided superior sequence coverage compared to the database search. PowerNovo2 demonstrates a distinct advantage in both the number of proteins with improved coverage and the total count of additionally identified peptides, underscoring the model’s efficacy in immunopeptidomics tasks.

We conducted a comparative analysis of PowerNovo2 against π-PrimeNovo, which represents a leading non-autoregressive model for de novo sequencing. Results from a standard database search using the SEQUEST algorithm, which identified 4,448 proteins and 27,611 peptides, served as the reference ground truth. The analysis demonstrated a significant performance advantage for PowerNovo2 over the competing non-autoregressive solution. PowerNovo2 successfully identified 1,540 reference proteins and recovered 4,003 peptide sequences, whereas π-PrimeNovo exhibited more conservative performance, identifying 816 proteins and 2,423 peptides. These metrics indicate a superior sensitivity of the proposed flow-based approach when processing complex immunopeptidomic data. The results of the protein coverage comparison are visualized in Fig 7.

For a subset of proteins in the HLA immunopeptidomics dataset, de novo sequencing was able to complement the results of traditional database searching by recovering additional peptides not identified by SEQUEST. Specifically, PowerNovo2 achieved higher sequence coverage than SEQUEST for 340 proteins, while π-PrimeNovo surpassed the database search for 215 proteins. For the proteins where PowerNovo2 outperformed SEQUEST, the mean relative increase in coverage was 169.6%, with a median increase of 100.0%. The maximum coverage gain observed was 4.8 percentage points, representing a 1400% relative increase for that particular protein. Collectively, PowerNovo2 recovered 786 additional peptides for this protein subset that were missed by the standard search. In comparison, for proteins where π-PrimeNovo outperformed SEQUEST, the mean coverage increase was 192.5%, though the total number of additionally recovered peptides was lower, at 552. It should be noted that the total number of proteins identified by de novo methods remains lower than that achieved by database searching; the results presented here reflect the complementary value of de novo sequencing for specific proteins rather than an overall superiority. These findings suggest that the hybrid variational architecture of PowerNovo2 can provide additional depth in immunopeptidomics profiling, particularly in the recovery of non-tryptic and modified sequences characteristic of HLA-presented peptides, which may fall outside the search space of conventional database approaches.

### Target–decoy benchmark

To further assess the discriminative power of the PowerNovo2 confidence scores, we performed a controlled target–decoy evaluation on the HLA benchmark dataset PXD055277. Following Tran et al. [14], decoy spectra were generated by perturbing real HLA class I MS/MS spectra through peak replacement at different noise levels, while preserving the precursor information and global peak distributions. For each original (“target”) spectrum, a matched “decoy” spectrum was constructed by randomly removing a specified fraction of peaks and replacing them with noise peaks drawn from the empirical peak distribution of the dataset. This procedure yields pairs of target and decoy spectra with nearly identical m/z and intensity statistics but disrupted fragment patterns, and is specifically designed to probe how well a de novo model can distinguish informative spectra from random matches (in S1 Appendix).

Using the public NovoBoard pipeline [14], we selected three decoy noise levels (50%, 60%, and 70% peak replacement) and applied PowerNovo2 and π‑PrimeNovo to the corresponding target and decoy spectrum sets. For each spectrum, both tools were run with identical preprocessing and no score filtering. For each noise setting, we then constructed score distributions for target and decoy peptide–spectrum matches (PSMs) and summarized them as histograms of the normalized confidence score in the range [0, 1].

This analysis serves two purposes. First, it provides a direct, database‑free view of how each model separates meaningful spectra from noise on a challenging HLA dataset, complementing peptide‑ and amino‑acid‑level accuracy metrics. Second, it illustrates the robustness of the scoring function under controlled spectrum degradation, which is critical for practical false discovery rate (FDR) control in de novo workflows.

**Fig 8:**
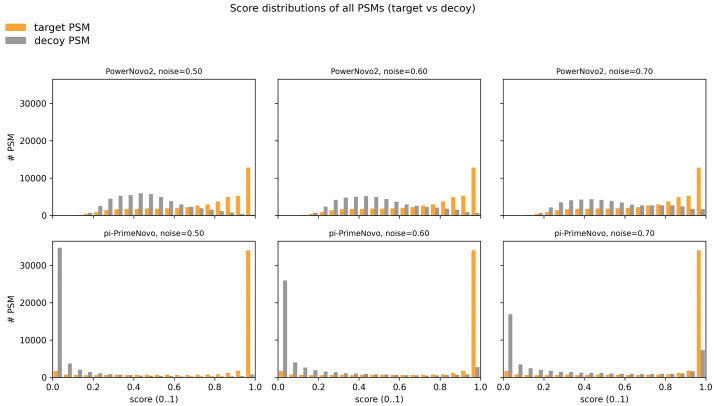
Score distributions of target and decoy PSMs for PowerNovo2 andπ‑PrimeNovo on NovoBoard HLA data. Score histograms for target (orange) and decoy (gray) peptide–spectrum matches (PSMs) obtained on the HLA benchmark dataset PXD055277 using the target–decoy spectrum framework of Tran et al. Each panel shows the distribution of normalized confidence scores (score01) in the [0, 1] interval. The first row corresponds to PowerNovo2, the second row to π‑PrimeNovo; columns represent decoy noise levels of 50%, 60%, and 70% peak replacement, respectively. For each original HLA MS/MS spectrum, a decoy spectrum was generated by randomly removing a fixed fraction of peaks and replacing them with noise peaks sampled from the empirical peak distribution, preserving global m/z and intensity statistics while disrupting informative fragment patterns. Both models were applied to all target and decoy spectra without score filtering, and all reported PSMs are included. The plots illustrate how well each model separates real spectra from noise as the degree of corruption increases. At moderate noise (50%), π‑PrimeNovo produces more high‑score target PSMs with a lower decoy fraction, while at higher noise (60–70%) PowerNovo2 yields a more conservative score distribution with fewer high‑score decoys, reflecting stronger robustness of its confidence calibration on degraded spectra.

Quantitatively, both PowerNovo2 and π‑PrimeNovo assign substantially higher scores to target PSMs than to decoy PSMs across all noise levels, confirming that their confidence scores carry meaningful information for FDR estimation (Fig 8). At moderate decoy noise (50%), π‑PrimeNovo shows a more aggressive separation between target and decoy PSMs: for example, at a score threshold of 0.7, π‑PrimeNovo retains 39,430 target PSMs and 2,231 decoy PSMs (decoy fraction ≈ 0.054), whereas PowerNovo2 retains 32,303 target PSMs and 5,959 decoy PSMs (decoy fraction ≈ 0.156). Even at a stringent threshold of 0.9, both models achieve a low decoy fraction (≈3%).

As the decoy noise increases to 60% and 70%, the relative behavior of the two models becomes more nuanced. At 60% peak replacement, π‑PrimeNovo still preserves more high‑score target PSMs than PowerNovo2, but the fraction of decoys among its high‑score PSMs grows faster. At the strict threshold 0.9, PowerNovo2 yields a decoy fraction of ≈0.078 compared to ≈0.091 for π‑PrimeNovo, indicating that PowerNovo2 becomes slightly more conservative on heavily perturbed spectra. At the highest noise level (70%), the target and decoy score distributions of both models collapse, as expected, but PowerNovo2 maintains lower relative scores for decoys, whereas π‑PrimeNovo still produces a small number of high‑score predictions on decoy spectra. Overall, these patterns suggest that π‑PrimeNovo emphasizes sensitivity and aggressively assigns high scores to a large fraction of targets, while PowerNovo2 adopts a more cautious scoring strategy that better suppresses spurious high‑confidence matches under severe spectrum corruption.

Taken together with our peptide‑ and amino‑acid‑level results, this target–decoy analysis highlights complementary strengths of the two non‑autoregressive models. π‑PrimeNovo is highly effective at recovering a large number of high‑score PSMs on clean or moderately noisy data, which is advantageous for large‑scale, high‑quality datasets. PowerNovo2, in contrast, offers a more conservative score calibration on degraded or noisy spectra, which is beneficial for controlling FDR in applications where spectrum quality is heterogeneous (e.g., immunopeptidomics, low‑abundance peptides, partially hydrolyzed samples). These properties are consistent with the design of PowerNovo2 as a flow‑based generative model with explicit uncertainty modeling and mass‑constrained post‑processing, which jointly promote robust discrimination between informative and noise‑dominated spectra.

### Performance speed of *de novo* services

A crucial aspect of *de novo* service evaluation is the assessment of processing speed, as this directly affects the practical implementation of these tools in proteomic workflows. The analysis of large-scale scientific and clinical datasets, often encompassing tens to hundreds of thousands of spectra, is frequently hampered by slow algorithms, which can create bottlenecks and extend the overall experimental duration.

**Fig 9:**
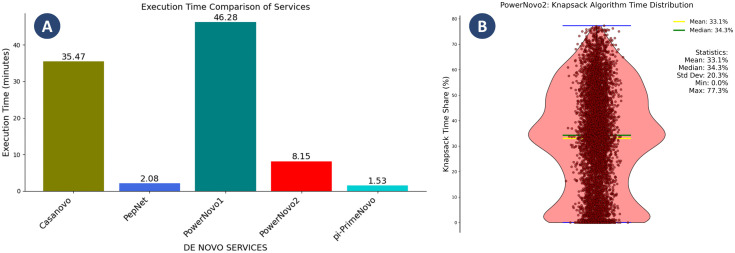
A. Processing speed for 10,000 spectra by de novo services: Casanovo v.4.3.0 (using beam search during decoding), PowerNovo v.1, and non-autoregressive models PepNet, PowerNovo2, π-PrimeNovo. Experiments were conducted on hardware running the Ubuntu 22.04 operating system, with the following hardware configuration: GPU NVIDIA GeForce GTX 1650 Ti with 4 GB of memory and CUDA version 12.8 support; CPU AMD Ryzen 7 4800H with 16 cores and a clock speed of 2.9 GHz; RAM 32 GB. Batch size = 16 for each model. The processing speed was measured in minutes. The time spent on preprocessing the mgf file with the spectrum, which is performed by all models, was not included. B. Distribution of knapsack algorithm execution time share in PowerNovo2 service. The violin plot illustrates the percentage of total prediction time consumed by the knapsack optimization algorithm across iterations. The yellow line indicates the mean value (33.1%), while the green line shows the median (34.3%). Individual data points (red circles) represent specific iteration measurements. The wide distribution (standard deviation = 20.3%) and broad range (0.0% to 77.3%) demonstrate significant variability in computational demands, with knapsack operations accounting for approximately one-third of total processing time on average.

This study presents an evaluation of the processing speed of the services under consideration using a single dataset and identical hardware conditions. Fig 9 illustrates the test findings. A test file including 10,000 spectra corresponding to peptides of different lengths was generated for the experiment. The specified file was compiled by random sampling from the NIST database, focusing on high-quality *H. sapiens* HCD spectra, primarily tryptic peptides without missed cleavages [15].

The experiments were performed on hardware with Ubuntu 22.04 operating system installed, in the following hardware configuration: NVIDIA GeForce GTX 1650 Ti GPU with 4 GB of memory, CUDA version 12.8 support; AMD Ryzen 7 4800H CPU with 16 cores and 2.9 GHz clock speed; RAM 32 GB. The batch size for each of the models used in the testing was fixed at 16. It should be noted that the comparative evaluation of PEAKS service performance was not conducted due to its belonging to closed commercial products operating exclusively on the Windows platform. Furthermore, the architectural and algorithmic aspects of PEAKS are inaccessible for analysis, thus precluding its inclusion in the comparative assessment of the models under review.

The comparative graphs above clearly indicate a marked advantage in data processing speed for non-autoregressive models over their autoregressive counterparts. In particular, the π-PrimeNovo model processes the benchmark set of 10,000 spectra in 1.53 minutes, outperforming the autoregressive Casanovo (35.47 minutes) and PowerNovo1 (46.28 minutes) by more than an order of magnitude. PowerNovo2 completes the same task in 8.15 minutes, which is approximately 4.35 times faster than Casanovo and approximately 5.68 times faster than PowerNovo1. This significant performance gap stems from the fundamental architectural differences between the model types. Autoregressive models generate peptide sequences iteratively, with each amino acid prediction contingent upon all previous ones, enforcing sequential computation that scales linearly with sequence length. In contrast, non-autoregressive models, including PowerNovo2, decode the entire sequence in a single, parallel forward pass through the network, dramatically reducing inference time.

The thorough performance analysis revealed the main bottleneck of PowerNovo2 to be the post-processing stage, in which peptide predictions are validated and corrected using a full knapsack optimization algorithm. As shown in Fig 9B, this step accounts for a mean of 33.1% and a median of 34.3% of total inference time, with considerable variability (standard deviation 20.3%, range 0.0%–77.3%) reflecting the dependence of combinatorial search complexity on sequence length and reconstruction ambiguity. It is important to note that the speed advantage of competing non-autoregressive models over PowerNovo2 stems from different architectural trade-offs rather than a fundamentally superior design. PepNet replaces full knapsack optimization with a simplified single-pass mass correction procedure, which reduces computational cost but also limits reconstruction accuracy for complex sequences. π-PrimeNovo implements an analogous mass-fitting algorithm natively on the GPU via CUDA, which eliminates the CPU-GPU transfer overhead at the cost of substantially increased deployment complexity, including custom C++ compilation and strict system dependency requirements. PowerNovo2, by contrast, prioritizes reconstruction correctness and deployment accessibility, being fully installable via standard pip without any compilation steps. Even with the current CPU-based knapsack implementation, PowerNovo2 remains 4.3 times faster than Casanovo and 5.6 times faster than PowerNovo1. A GPU-accelerated CUDA implementation of the knapsack algorithm is planned as a future optimization and is expected to further reduce the performance gap with π-PrimeNovo.

### Ablation study of model architecture

To comprehensively evaluate the architectural design choices of PowerNovo2, we conducted an extensive ablation study examining the impact of three critical components: (1) the knapsack-based sequence optimization algorithm, (2) the latent space noise distribution characteristics, and (3) the choice of probability distribution for latent variable modeling.

The study was performed on a standardized test set containing 1000 peptide sequences. The complete PowerNovo2 model (with knapsack optimization and normal distribution latent noise) demonstrated superior performance with 84.92% accuracy, establishing the reference benchmark for architectural comparisons. Removal of the knapsack-based optimization algorithm resulted in significant performance degradation to 81.92% (Δ = -3.00%), demonstrating the algorithm’s essential role in sequence reconstruction and validation.

The model showed remarkable sensitivity to latent space distribution choices. The uniform distribution configuration achieved 68.13% accuracy (Δ = -16.78% from baseline), highlighting the importance of normal distribution assumptions for optimal performance.

Systematic variation of latent noise standard deviation revealed a pronounced dependence of model performance on regularization level. We observed a consistent decrease in prediction accuracy with increasing standard deviation: from 80.23% at σ = 0.1 to 73.54% at σ = 3.0, corresponding to a performance reduction of 6.69 percentage points. This pattern indicates quality degradation in modeling with excessive latent space noise, confirming the importance of controlled regularization for preserving model predictive capabilities.

**Fig 10:**
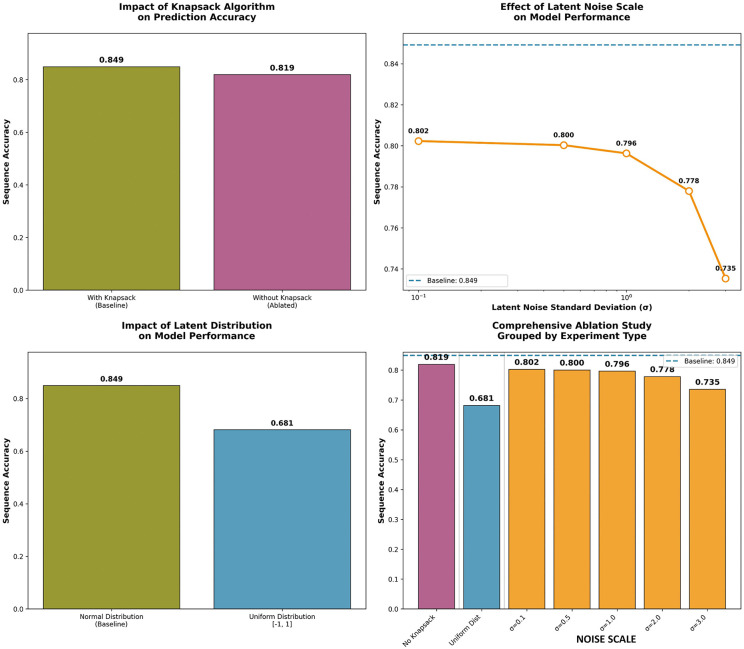
Comparative visualization of ablation study results, grouped by experimental category (knapsack optimization, distribution type, and noise scale). The dashed line indicates baseline performance for reference. All ablated configurations show measurable performance degradation, confirming the importance of each architectural component.

The complete results of our ablation study are presented in Fig 10, showing comparative performance across all experimental conditions. The baseline configuration consistently outperformed all ablated variants, confirming the synergistic integration of architectural components in PowerNovo2.

These findings provide empirical validation for several key design decisions in PowerNovo2: the knapsack algorithm provides essential combinatorial optimization for sequence validation; normal distribution assumptions in latent space modeling outperform uniform alternatives; the integrated architecture demonstrates robustness across varying experimental conditions.

The ablation study confirms that PowerNovo2’s performance stems from the carefully balanced integration of its components rather than any single architectural element, providing a solid foundation for proteomic applications requiring high-accuracy de novo peptide sequencing.

## Discussion

PowerNovo2 introduces a generative flow-based framework for non-autoregressive de novo peptide sequencing, marking a significant departure from traditional autoregressive models by enabling parallel token prediction while explicitly modeling uncertainties through latent variables. This architecture leverages variational inference and invertible transformations to capture complex dependencies in MS/MS spectra, addressing key limitations such as error propagation and sequential decoding bottlenecks. Our comprehensive benchmarking, including direct comparisons with state-of-the-art tools like Casanovo, PepNet, and the recently published π-PrimeNovo (Nature Communications, 2025), demonstrates that PowerNovo2 achieves competitive performance in accuracy while offering substantially faster inference: on a standardized test set of 10,000 spectra, PowerNovo2 completed inference in 8 minutes 15 seconds, compared to 35 minutes 47 seconds for Casanovo (4.3-fold speedup) and 46 minutes 28 seconds for PowerNovo1 (5.6-fold speedup). While π-PrimeNovo often outperforms PowerNovo2 in overall peptide-level accuracy across standard datasets—achieving up to 10–15% higher precision on clean, tryptic peptides from datasets like Nine-Species and MassIVE-KB – our model excels in niche scenarios that highlight the advantages of its probabilistic design. Quantitatively, PowerNovo2 shows 20–30% higher accuracy on peptides longer than 30 amino acids (Figs 2 and 3), where π-PrimeNovo’s performance drops by ~15% relative to shorter sequences due to challenges in modeling extended dependencies. Similarly, on low-resolution NIST spectra with partial hydrolysis and noise, PowerNovo2 maintains 10–20% higher peptide identity rates at >90% sequence similarity thresholds (Fig 5), reflecting its robust uncertainty handling. In immunopeptidomics applications using the PXD055277 HLA dataset, PowerNovo2 identifies 1,540 proteins and 4,003 peptides, surpassing π-PrimeNovo’s 816 proteins and 2,423 peptides, and provides superior protein coverage gains (mean 169.6% relative increase over SEQUEST for 340 proteins, with 786 additional peptides recovered; Fig 7). These strengths are further evidenced by the UpSet analysis (Fig 4), where PowerNovo2 uniquely identifies 10,625 peptides (6% of 161,969 total), over four times more than its closest competitor.

The target-decoy benchmark on perturbed HLA spectra reinforces these complementary profiles. At moderate noise (50% peak replacement), π-PrimeNovo retains more high-score target PSMs (e.g., 39,430 at threshold 0.7 with decoy fraction ~0.054) compared to PowerNovo2 (32,303 with ~0.156), prioritizing sensitivity. However, at higher noise levels (60–70%), PowerNovo2’s conservative scoring yields lower decoy fractions (e.g., ~ 0.078 at 0.9 threshold vs. ~ 0.091 for π-PrimeNovo), better suppressing false positives in degraded data (Fig 8). This behavior aligns with PowerNovo2’s explicit latent modeling, which provides calibrated confidence under uncertainty, versus π-PrimeNovo’s aggressive assignments on cleaner spectra.

A deeper examination of architectural trade-offs illuminates these differences. π-PrimeNovo’s mass-controlled decoding integrates precursor mass constraints directly into the Transformer layers via CUDA-optimized inference, enabling rapid, deterministic filtering that maximizes exact matches on high-quality data (up to 10 × speed over autoregressives). This approach excels in scalability for large metaproteomics datasets but can overfit to mass priors, potentially amplifying errors in noisy or atypical spectra where multimodal uncertainties arise. In contrast, PowerNovo2’s flow-based latent modeling decouples density estimation from decoding, using invertible transformations (e.g., actnorm, multi-head linear layers, affine coupling) to represent complex joint distributions without assuming conditional independence. This yields robust generalization but introduces computational overhead in post-processing (e.g., knapsack optimization for mass consistency), as confirmed by our ablation study: removing knapsack drops accuracy by 3% (from 84.92% baseline to 81.92%), while uniform latent distributions reduce it by 16.78% (Fig 10). Thus, PowerNovo2 trades some raw speed for enhanced reliability in heterogeneous conditions, offering a complementary tool where π-PrimeNovo’s determinism may falter.

These results underscore PowerNovo2’s value as the first flow-based de novo sequencer, particularly for applications like immunopeptidomics, low-abundance proteomics, and non-model organisms. Future work could enhance its efficiency by GPU-accelerating knapsack solving, as our analysis identifies this as the primary bottleneck. By open-sourcing PowerNovo2 with pre-trained weights and a Python package, we aim to foster further innovation in probabilistic AI for proteomics, potentially hybridizing flow-based and mass-controlled strategies for even broader applicability.

## Methods

### Strategy for our non-autoregressive modeling

*De novo* sequencing of mass spectrometry data is largely performed via autoregressive sequence-to-sequence neural network models, which factorize the joint probability of the output sequence into the product of conditional probabilities for each successive token. The calculation of each probability incorporates the input sequence and previously generated tokens. This can be represented as follows:


$$\begin{array}{c} P_{\theta }(y|x)=\;\prod_{i=\text{1}}^{\text{T}}P_{\theta }\left(y_{t}\right|y_{<t},\;x), \end{array}$$ (1)

where *θ* are the model parameters, with each factor $\left(y_{t}\right|y_{<t},\;x)$ defined by an approximation function implemented by neural networks such as Transformer (Casanovo [5], PowerNovo v.1 [6] models) or LSTM (DeepNovo) [16].

This factorization converts the intricate problem of joint estimation into a more tractable series of multi-class classification subproblems, facilitating the prediction of *y*_*t*_ based on the preceding tokens.

However, from a modeling standpoint, the optimality of assuming linear factorization in either a left-to-right or a right-to-left sequence is questionable. This concern is due to the fact that the linear processing of output tokens, inherent in the generation process, may not fully capture the intricate dependencies within complex output sequences. Furthermore, memory-constrained search algorithms, including beam and greedy search, hinder parallelization, thus limiting model performance.

Within the last two years, studies on non-autoregressive methods for generating peptide sequences from mass spectrometric data have been published [8,17]. Non-autoregressive models are designed to model directly the complete joint distribution of $P_{\theta }(y|x)$, weakening the dependencies on historical decoding data during the generation process. A straightforward approach to non-autoregressive model construction assumes of mutual independence among output sequence tokens. The efficiency of this model is significantly lower than that of traditional autoregressive methods due to strong conditional dependencies among the output variables. This limitation can be overcome by introducing a latent variable, z, to model these conditional dependencies. Formally, this can be expressed as follows:


$$\begin{array}{c} P_{\theta }\left(y|x\right)=\;\int_{z}P_{\theta }\left(y|z,\;x\right)p_{\theta }\left(z|x\right)dz, \end{array}$$ (2)

where $p_{\theta }\left(z|x\right)$ is the a priori distribution on the latent variable z, and $P_{\theta }\left(y|z,\;x\right)$ is the generating distribution (decoder). Non-autoregressive generation, in this case, is achieved through the following independence assumption in the decoding process:


$$\begin{array}{c} P_{\theta }\left(y|z,\;x\right)=\;\prod_{t=\text{1}}^{T}P_{\theta }\left(y_{t}|z,\;x\right) \end{array}$$ (3)

Our PowerNovo2 solution models the a priori distribution $P_{\theta }\left(y|z,\;x\right)\;$ using a mathematical apparatus called normalized or generative flow [18]. Generative flows are a powerful tool for modeling complex posterior distributions in the context of variational inference.

### Generative flows

Flow-based generative models transform a simple distribution (e.g., Gaussian) into a complex one through reversible transformations. These transformations allow efficient Jacobian determinant computation and improve posterior approximations, achieving better alignment with the true posterior and boosting model performance.

### Reversible transformations

For the complex a priori distribution *z* to be modeled, we introduce a set of latent variables ϑ∈Υwith a simple a priori distribution $p_{\gamma }(\vartheta )$. Then, a bijection function f:Z→ Υ and $g=f^{-\text{1}}$ is defined by which to describe a generative process over the variables *z*:


$$\begin{array}{c} \vartheta \sim \;p_{\gamma }(\vartheta )\; \end{array}$$

$$\begin{array}{c} \;z=g_{\theta }(\vartheta ) \end{array}$$ (4)

The crucial aspect of this approach is that, given a bijection function, the variable change formula defines the model distribution on z∈Z as follows:


$$\begin{array}{c} p_{\theta }(z)=\;p_{\gamma }\left(f_{\theta }(z)\right)\left|\text{det}\left(\frac{\partial f_{\theta }(z)}{\;\partial z}\right)\right| \end{array}$$ (5)

where $\frac{\partial f_{\theta }(z)}{\partial z}$ represents the Jacobian of the function $f_{\theta }$ at the point z.

Equation 5 provides a way to compute the complex distribution density *z* by computing the simple distribution density u and the Jacobian of the transformation from *z* to ϑ. The efficiency of a flow-based model is commonly improved by using certain types of $f_{\theta }$ transformations, where both the inverse functions $g_{\theta }$ and the determinants of the Jacobians are easy to compute.

### Maximizing the variation lower bound

Maximum likelihood estimation (MLE) dictates that model parameters should be selected to maximize the likelihood of the observed data. Therefore, we aim to minimize the negative log-likelihood function, which is analogous to maximizing the logarithmic likelihood of the model. Employing logarithmic transformations of probability values facilitates mathematical computations and mitigates numerical instability inherent in the calculation of extremely small probabilities. Therefore, a formal representation of this problem can be presented as follows:


$$\begin{array}{c} \underset{\theta \in \Theta }{\text{min}}\frac{\text{1}}{N}\sum_{i=\text{1}}^{N}-\text{log}P_{\theta }\left(x_{i}|y_{i}\right), \end{array}$$ (6)

where θ represents the model parameters, Θ is the parameter space, N is the total number of observations, and $P_{\theta }\left(x_{i}|y_{i}\right)$ denotes the conditional probability of the observed value xi, provided by the parameters θ and the corresponding output values yi. $\{P_{\theta }\left(x_{i}|y_{i}\right)\overset{N}{\underset{i=\text{1}}{\}}}$ can also be considered as a training set.

The main challenge is that the computation of the likelihood $P_{\theta }\left(x_{i}|y_{i}\right)$ is hampered by the presence of latent variables *z*. In this case, the expression can be represented as:


$$\begin{array}{c} P_{\theta }\left(y|x\right)=\;\int P_{\theta }\left(y,\;z|x\right)dz, \end{array}$$ (7)

where the limit integration of the variable *z* is difficult to perform numerically, especially in high-dimensional spaces.

The computational complexities of limit integration are mitigated through the application of variational inference [19]. A solution to this problem is proposed by variational inference, which introduces a parametric model $q_{\phi }\left(z|y,\;x\right)$ approximating the true posterior distribution, with *ϕ* being the parameters of this approximation. This model serves to approximate the integral by sampling individual examples of the variable *z*. Given the above, the aim is to maximize the Evidence Lower Bound (ELBO), which provides an approximation of the true likelihood, in the following manner:


$$\begin{array}{c} \text{log}P\left(y|x\right)\;\geq E_{q_{\phi }\left(z|y,\;x\right)}\left(\text{log}P_{\theta }(y|z,\;x)\right)-KL(q_{\phi }\left(z|y,\;x\right)|\left|p_{\theta }\left(z|x\right)\right), \end{array}$$ (8)

where $E_{q_{\phi }\left(z|y,\;x\right)}\left(\text{log}P_{\theta }(y|z,\;x)\right)$ is the reconstruction error, which assesses how well the model can reconstruct the data from the latent variables *z*, and *KL* represents the Kullback-Leibler divergence, which measures the distance between the approximated distribution and the true posterior distribution.

The training process refines model parameters *ϕ* and decoder parameters *θ* to achieve efficient computation and approximation of complex posterior distributions.

### Model architecture

**Fig 11:**
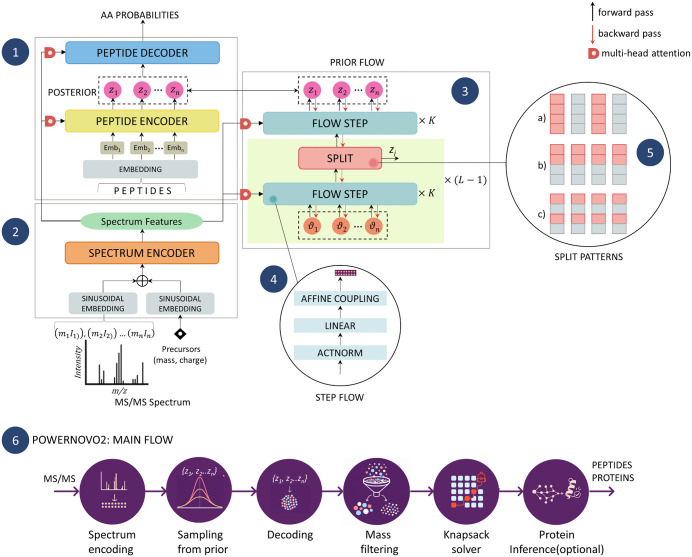
Architecture of PowerNovo2. Architecture includes the encoder (2), the decoder and the posterior networks (1), and prior flow (3, 4, 5). The general presentation of the steps of the process is provided in (6).

A review of the architecture (Fig 11) and the training procedure is presented before the detailed description of each PowerNovo2 module. PowerNovo2 processes mass spectrometry data, generating a latent representation termed a spectral “embedding”. Subsequent to the transformations depicted in Fig 11, the resultant datasets comprise peptides, their cognate proteins, and associated confidence coefficients for each amino acid residue and the peptides themselves.

This generative flow model architecture, an advanced method for modeling data distributions, is founded upon the work of Durk P. Kingma and Prafulla Dhariwal on generative flow with invertible 1x1 convolutions [20]. This architecture is centered on generative and normalizing flows, enabling the efficient acquisition and prediction of intricate distributions via the successive application of reversible transformations to the data.

### Training process

The training process optimizes the model parameters within the variational paradigm described in the section on maximizing the variational lower bound. The first step involves generating the samples of latent variables *z* from the current posterior distribution $q_{\phi }\left(z|y,\;x\right)$. The resulting *z* values are then passed, together with the spectral embedding, to both the decoder network and the prior flow to compute the conditional probability $P_{\theta }(y|z,\;x)$ and $p_{\theta }\left(z|x\right)\;$ required for the optimization of the lower variation estimator (ELBO), as shown in Eq. 8.

### Peptide sequence prediction process

The prediction process initially generates latent variables *z* extracted from the *prior flow* by executing the generative process defined by Eq. 5. The spectral “embeddings” produced by the encoder function as conditional inputs at this point in the process. Subsequently, the decoder unit integrates the sampled *z* variables and spectral “embeddings” to generate the target sequence *y* from its probability distribution $P_{\theta }(y|z,\;x)$. This methodology effectively leverages probabilistic models and the structured relationships between latent variables *z* and input data to facilitate robust learning and generation.

### Peptide molecular weight control

In order to improve the accuracy of the generated peptide sequences, we must focus on the molecular mass measured via a mass spectrometric detector. A crucial requirement is that the molecular mass of the peptide should fall within the interval [m − σ, m + σ], with m representing the measured precursor ion mass and σ denotes the allowable relative mass measurement error (typically 10–20 ppm for high-resolution mass spectrometers).

This requirement is satisfied by solving an optimization problem akin to the 0/1 Knapsack Problem. This problem requires determining the set of elements (amino acids) that best fills a “knapsack” subject to a weight constraint. The importance of each element is determined by its predicted logarithmic probability.

### Protein inference

Peptide detection is a key step in protein identification, where peptides are matched to user-defined FASTA databases and assembled into amino acid sequences using the ALPS peptide assembler [21]. This process significantly enhances sensitivity by reconstructing partial or incomplete proteins from fragmented data, thereby improving overall proteome coverage. Aggregation of peptides into contigs is particularly beneficial when studying complex biological systems such as monoclonal antibodies (mAbs), enabling more accurate reconstruction of their primary structure even with limited experimental evidence.

The process consists of three stages: (1) peptides are aggregated into contigs using the De Bruijn ALPS assembler, yielding extended sequences; (2) contigs are matched to proteins in a user-defined FASTA database via the fast and efficient npyseach library, producing contig-protein mappings; (3) Protein Inference is performed using the REPRISAL^18^ algorithm to determine target proteins. This streamlined approach ensures more accurate protein identification from peptide data.

### Modules

#### Spectrum encoder.

A traditional Transformer encoder, previously implemented as Casanovo [5] and PowerNovo v.1 [6], underpins the spectrum encoding model. The encoder processes spectral peaks, each characterized by a Mass-to-Charge ratio (m/z) and a monotonically related intensity $S=\{(m_{i},\;I_{i})\overset{N}{\underset{i=\text{1}}{\}}}$. For representing each m/z value, we use a sinusoidal position-encoding function, using which the m/z values are mapped into d-dimensional vectors *f:*


$$\begin{array}{c} f_{i}=\;\left\{ \begin{array}{c} \text{sin}(\frac{m_{i}}{\frac{\lambda_{max}}{\lambda_{min}}(\frac{\lambda_{max}}{\text{2}\pi }{)}^{\frac{\text{2}i}{d\;}}\;}),\;for\;i\;\leq \;\frac{d}{\text{2}}\\ \text{c}os(\frac{m_{i}}{\frac{\lambda_{max}}{\lambda_{min}}(\frac{\lambda_{max}}{\text{2}\pi }{)}^{\frac{\text{2}i}{d\;}}\;}),\;for\;i>\;\frac{d}{\text{2}} \end{array}\right.\; \end{array}$$ (9)

where i denotes the position in the d-dimensional vector, with λ_max_ = 10,000 and λ_min_ = 0.001 indicating the wavelengths for this coding from 0.001 to 10,000 m/z. The spectrum intensity values are mapped into a d-dimensional vector using a linear layer of the encoder network and summarized with the vector *f*.

### Posterior neural network

#### Generation of latent variables.

Our solution uses latent variables *z*, structured as a sequence of continuous random vectors *z= {z*_*1*_*…z*_*T*_*}*, each vector possessing a dimensionality identical to that of the target peptide sequence. Every element, denoted *z*_*t*_, constitutes a vector of dimension *d*_*z*_, where *d*_*z*_ signifies the dimensionality of the latent space.

The architecture employs two fundamental components to process peptide sequences:

1. A trainable amino acid embedding layer that converts discrete residue tokens into continuous vector representations
2. A transformer-based peptide encoder that captures contextual relationships between residues through self-attention mechanisms

These components collectively generate the parameters for the diagonal normal distribution:


$$\begin{array}{c} q_{\phi }\left(z|y,\;x\right)=\;\prod_{t=\text{1}}^{T}N\left[z_{t}|\mu_{t}(x,\;y),\;\sigma^{\text{2}}(x,\;y)\right],\; \end{array}$$ (10)

where $\mu_{t}(x,\;y)\;$ is the value defining the mean of the distribution at each step t, and $\sigma^{\text{2}}(x,\;y)$ is the dispersion. Both values (mean and variance) are computed in our solution using a neural network of Transformer architecture.

#### Modeling uncertainty and token dependencies in the sequence.

The latent variable *z* is introduced to model uncertainty and capture contextual interdependencies in peptide sequences. However, without precautions, the model may converge to a trivial solution where *z*_*t*_ reflects only the current token, limiting generalization. To avoid this, a dropout mechanism randomly removes 33% of tokens during posterior calculation, forcing the model to rely on both *z*_*t*_ and the broader context. This approach is similar to masked language modeling techniques^15^.

### Prior neural network (generative flow)

The model is architecturally organized as a hierarchy of flow steps. This enables the model to process data across various scales, thus facilitating the identification and utilization of diverse interdependencies among data elements. Each flow step incorporates three elementary flow types: actnorm, invertible multi-head linear transformations, and coupling layers. A defining characteristic of elementary streams is their reversibility, ensuring lossless recovery of the original data from its transformed state. Consequently, the calculation of the transformation Jacobian is significantly simplified, which is necessary for accurate and efficient computation of logarithms of determinants. Accurate log determinant computation is crucial for probabilistic data modeling, as it directly impacts the calculation of distribution density under any Boolean representation.

#### Activation normalization (actnorm).

Activation normalization (actnorm), an alternative to the prevalent batch normalization method, was developed to enhance model training for image processing [20]. Actnorm employs an affine transformation, parameterized by input-specific scale *(s)* and bias (*b*) values for each feature, to normalize activations. The formula governing this transformation is presented below:


$$\begin{array}{c} \overset{´}{z_{t}}=\;s\;⨀\;z_{t\;}\;+b, \end{array}$$ (11)

Where *z*_*t*_ *и z*_*t*_*′* are input and output activation tensors of dimension *[T × d*_*z*_*]*, with T denoting the time dimension and а *d*_*z*_ the number of features, s denoting a scaling parameter (vector) that is applied postcomponentially and b a biasing parameter (vector).

The initialization of the parameters *s* and *b* is performed in such a way that in the initial mini data set, the transformed activations *z′* have zero mean and unit variance. This allows for stable training, eliminating the need to compute statistics across mini-batches as in batch normalization.

Given the independent scaling and shifting nature of each component, the Jacobian matrix of this transformation assumes a diagonal form; the diagonal entries are precisely the values of *s*. This facilitates the calculation of the logarithmic determinant.

The logarithm of the determinant of the Jacobi matrix from this transformation is equal to the product of the logarithms of the absolute values of the diagonal elements multiplied by the dimensionality of the time dimension *T*, since all components at each time point undergo the same transformation:


$$\begin{array}{c} \text{log}|\text{det }J|=T\cdot \;\sum \text{log}|s|, \end{array}$$ (12)

where *J* is the Jacobi matrix of the transformation, *T* is the number of elements in the sequence, and ∑log|s|   is the sum of the logarithms of the absolute values of the scaling parameters for each component.

#### Invertible multi-head linear layers.

Our reversible layer implementation draws upon the research of Kingma and Dhariwal [20] concerning generative flow and invertible 1x1 convolutions. Their work describes a trainable reversible convolutional network utilizing one-dimensional convolutions for processing two-dimensional images. The subsequent transformation is applied to facilitate the processing of sequential mass spectral data:


$$\begin{array}{c} {\overset{´}{z}}_{t}=z_{t\;}\text{W}, \end{array}$$ (13)

where *W* denotes a matrix of weights of dimension *[d*_*z*_ *× d*_*z*_*].*

The logarithm of the determinant of the corresponding linear transformation is computed as:


$$\begin{array}{c} \text{log}\left|\text{det}\left(\;\frac{\partial \text{linear}(z;\;W)}{\partial z}\right)\right|=T\cdot \;\text{log}\left|\text{det}(W)\right|, \end{array}$$ (14)

where *T* represents the length of the spectrum sequence.

The computational complexity of finding det(*W*) is $\text{0}\left(\overset{\text{3}}{\underset{z}{d}}\right)$, affecting the efficiency negatively because the dimensionality *d*_*z*_ in our solution is large enough to be 512. To mitigate the computational burden, we employ a multi-stage reversible linear layer. This layer initially partitions each feature vector of dimensionality *d*_*z*_ into *h* heads, each of the size *d*_*h*_ *= d*_*z*_*/h*. Then, the linear transformation by Equation 14 is applied to each head with a weight matrix of size *d*_*h*_ *× d*_*h*_, significantly reducing the computational resource requirements. For head splitting, one flow step contains one linear layer with a row or post-column partitioning scheme, and these steps with different linear layers are interleaved.

#### Affine coupling layers.

In order to model the dependency between time steps, we apply the affine linking layers. These layers are realized as follows: two parts, denoted as *z*_*a*_ and *z*_*b*_, are extracted from the input tensor *z* using the partition function *f*_*split*_*.* Then, a transformation is performed using the following scheme:


$$\begin{array}{c} \begin{array}{c} z_{a},\;z_{b}=\;f_{split}(z)\\ \overset{´}{z_{a}}=z_{a}\\ \overset{´}{z_{b}}={net}_{\text{1}}\left(z_{a},\;x\right)⊙z_{b}+{net}_{\text{2}}\;\left(z_{a},x\right)\\ \overset{´}{z}=\;f_{concact}\left(\overset{´}{z_{a}},\overset{´}{z_{b}}\right),\; \end{array} \end{array}$$ (15)

where ${net}_{\text{1}}\left(z_{a},\;x\right)\text{ and }{net}_{\text{2}}\;\left(z_{a},x\right)$ are the outputs of two neural networks implemented using a single decoder layer of the traditional transformer framework, the function *f*_*split*_ divides the input tensor z into two equal parts, and the function *f*_*concat*_ performs the corresponding inverse operation of combining the parts.

Three partitioning function types are used in this architecture, with the choice depending on the dimensionality and the pattern of partitioning. The first type of partitioning groups the *z* values time dimension through a single index, thereby enabling the representation of interactions between different time points. The second and third partitioning methods operate along the feature dimension, exhibiting continuous and alternating patterns respectively. Fig 11(5) illustrates the example templates. For each type of partitioning, *z*_*a*_ and *z*_*b*_ are interleaved to increase the flexibility of the partitioning function. Different types of affine binding layers are also interleaved in the flow, as depicted in Fig 11(4).

### Decoding

#### Decoder.

The PowerNovo2 decoder block operates on an input sequence of latent variables *z*, feeding them through the multiple layers of a Transformer-based decoder network. This is followed by the autonomous prediction of each output token in the peptide sequence. This approach is distinguished from traditional autoregressive Transformer decoders by its exclusion of causal masking, thus enabling the processing of future tokens. This enables the model to operate in a completely non-autoregressive manner.

#### Peptide sequence length.

Autoregressive seq2seq models typically determine sequence length using a stop token. In contrast, PowerNovo2 requires parallel sequence prediction, needing the length beforehand to generate the latent variable sequence z. Instead of exact length prediction, it estimates the difference between the mean peptide length (48 amino acids) and the target length using a classifier predicting values from -24–24. This involves max pooling on input sequence vectors, processing through a linear layer, and applying softmax. The classifier and model are trained together.

#### Decoding process.

This section details a novel decoding methodology developed to address the unique challenges of mass spectrometric data and peptide sequence analysis. This approach permits the efficient selection of appropriate decoding candidates, resulting in optimized computational resource utilization. Fig 11 illustrates the decoding process, which comprises the following stages:

1. **Initial sample selection**: The first step involves selecting *k* samples of each sequence from the a priori distribution, where *k* is determined by the parameters of the model settings (default *k* = 5).
2. **Precursor mass filtering**: The sample selection is predicated on peptide mass falling within a defined precursor mass tolerance. Samples meeting this condition are immediately identified as final results and are not subjected to further stages of analysis, resulting in reduced computational burden.
3. **Probability maximization**: The selection of samples is based on maximizing the sum of positional probabilities within the remaining sample set.
4. **Optimization using the “knapsack” algorithm**: For the selected samples, the peptide mass optimization problem is solved using the “knapsack” algorithm (see the Knapsack Solver Section).

#### Knapsack solver.

For peptide mass analysis, the knapsack problem is solved using the CyLP library, a high-performance tool providing functions for linear and integer optimization [22]. This problem requires that the total mass of the amino acid residues in the peptides approximate the precursor mass, within the allowed tolerance. The optimization problem is formulated as follows:

1. **Target function:** The objective is to maximize the sum of the logarithms of the probabilities of observing the neural network outputs for a given peptide sequence S. This can be written as:

$$\begin{array}{c} maximize\;\sum_{i=\text{1}}^{T}\text{log}P\left(y_{i}\;\right|S) \end{array}$$ (16)

1. **Limitations:** The limitations relate to the cumulative weight of the elements that constitute the decoded peptide sequence. The total weight should be constrained to the interval [*L, U*].

Let us define Γ(y) as the set of all elements associated with the sequence *y*. For each element let *w*(*a*_*j*_) denote its weight (mass of an amino acid residue). Then, the limitation is formulated as follows:


$$\begin{array}{c} L\leq \;\sum_{a_{j\;}\in \;\Gamma (y)}w\left(a_{j}\right)\leq U,\; \end{array}$$ (17)

where *L = m – σ* и *U = m + σ*, with m being the mass of the precursor and *σ* being the error showing the tolerance limits of the deviations.

### Hyperparameters

Details of the datasets used for training and validation of the model, as well as the configuration of the model’s hyperparameters, are provided in S1 Appendix.

## Supporting information

S1 Appendix Figs A to B, Tables A to C, Hyperparameters, Target–decoy dataset. (DOCX)
