# Supplementary material for: PowerNovo2: A generative flow-based approach to non-autoregressive de novo peptide sequencing
Source: PLoS Comput Biol. 2026 May 20;22(5):e1014298. doi: 10.1371/journal.pcbi.1014298 (PMC13215612; doi:10.1371/journal.pcbi.1014298)
Supplement: S1 Appendix — (DOCX) [file pcbi.1014298.s001.docx]

S1 Appendix for

**PowerNovo2: A generative flow-based approach to non-autoregressive *de novo* peptide sequencing**

Denis V Petrovskiy^1^, Kirill S Nikolsky^1^, Vladimir R Rudnev^1^, Liudmila I Kulikova^1^, Tatiana V Butkova^1^*, Kristina A Malsagova^1^, Arthur T Kopylov^1^, Anna L Kaysheva^1^*

1. *Institute of Biomedical Chemistry, Moscow, Russia*

[*t.butkova@gmail.com*](mailto:t.butkova@gmail.com)*,* [*kaysheva1@gmail.com*](mailto:kaysheva1@gmail.com)

**Contents**

Hyperparameters

Table A

Table B

Figure A

Figure B

Table C

Target–decoy dataset

References

### ***Hyperparameters***

The encoder, decoder, and posterior c networks were implemented using layers of the classic transformer framework[1]. The spectrum encoder and decoder comprise 8 layers and 8 attention heads, d_model_/d_hidden_ = 256/512, the posterior networks comprise 8 layers and 8 attention heads.

The model was trained for 192 epochs with a mini-batch size of 256. The parameter optimization process was performed using the AdamW β= (0.9,0.999), ϵ=1e−8. The initial learning rate was set at 5e^−4^ with a subsequent decrease to 3e ^-6^. The KL error weight varied linearly from 0 to 1 over 32 epochs, which is necessary for more stable training of encoder, decoder, and posterior networks.

**Table A.** Training and test dataset.

| SOURCE | SPECIES | PRECURSORS | DESCRIPTION | Link |
| --- | --- | --- | --- | --- |
| TRAIN DATASET  Massive-KB  Spectral library v2 - full releases  KB 2.0.15 | H.sapiens | [5,948,126](https://massive.ucsd.edu/ProteoSAFe/result.jsp?task=e33a302ea7e94422bf2b122260d22cc6&view=ambiguity_library_view_split) | Human peptide spectral library constructed from 1.25 billion spectra from in vivo proteomics and synthetic peptide experiments, including spectra from multiple data types. Use to obtain the best evidence of peptide fragmentation for all peptide variants across all included datasets. | https://massive.ucsd.edu/ProteoSAFe/status.jsp?task=3cac03860ff7453a821332ab4cff20f4 |
| TEST DATASET  NIST | H.sapiens | 60, 000 | High- and medium-quality spectra with semi-tryptic peptides.  (60,000 random samples). | https://chemdata.nist.gov/dokuwiki/doku.php?id=peptidew:lib:humanhcd20160503 |

**Table B.** Validation datasets

| SOURCE | SPECIES | PRECURSORS | DESCRIPTION | Link |
| --- | --- | --- | --- | --- |
| Massive-KB  MSV000090982 | - Apis-mellifera - Bacillus-subtilis - Candidatus-endoloripes - H.-sapiens - Methanosarcina-mazei - Mus-musculus - Saccharomyces-cerevisiae - Solanum-lycopersicum - Vigna-mungo | 2,800,000 | De novo nine-species benchmark | https://data.niaid.nih.gov/resources?id=massive_msv000090982 |
| NIST | H.sapiens | 10,000 | Human Ion Trap Library.  10,000 samples were used for testing. | https://chemdata.nist.gov/dokuwiki/doku.php?id=peptidew:lib:human20140529 |
| NIST | H.sapiens | 30, 000 | Consensus Human HCD Libraries.  The dataset consists of three parts:   1. high-quality spectra, mostly tryptic peptides without missed cleavages; 2. medium-quality spectra, mostly tryptic peptides with missed cleavages; 3. high- and medium-quality spectra, mostly semi-tryptic peptides.   10,000 samples from each part were used for testing, excluding peptides that were utilized in the test dataset. | https://chemdata.nist.gov/dokuwiki/doku.php?id=peptidew:lib:humanhcd20160503 |
| NIST | H.sapiens  (hair) | 2,240 | Human Hair Peptide Spectral Library (Fusion Lumos – HCD) | https://chemdata.nist.gov/dokuwiki/doku.php?id=peptidew:lib:human_hair_selected_with_gvps_passed |
| NIST | H.sapiens | 10, 000 | Human Phosphopeptide Spectral Library (Orbitrap -HCD). 10, 000 samples were used for testing. | https://chemdata.nist.gov/dokuwiki/doku.php?id=peptidew:lib:human_hair_selected_with_gvps_passed |
| Massive-KB MassIVE-KB v2.0.15: Nontryptic only | H.sapiens | 10, 000 | MassIVE-KB v2, MS/MS spectra of peptides from proteomics experiments digested with various different enzymes. 10,000 samples were used for testing. | https://massive.ucsd.edu/ProteoSAFe/static/massive-kb-libraries.jsp |
| NIST | M.musculus | 10,026 | Mouse HCD Library. | https://chemdata.nist.gov/dokuwiki/doku.php?id=peptidew:clib:mousehcd_selected20141124 |
| NIST | M.musculus | 10,000 | Mouse Ion Trap Library. 10, 000 samples were used for testing. | https://chemdata.nist.gov/dokuwiki/doku.php?id=peptidew:lib:mouse20130520 |
| NIST | E. coli | 10,000 | E Coli Ion trap library.  10, 000 samples were used for testing. | https://chemdata.nist.gov/dokuwiki/doku.php?id=peptidew:lib:e_coli |
| NIST | Yeast  (S.cerevesiae) | 10,000 | Yeast Ion Trap Library. 10,000 samles were used for testing. | https://chemdata.nist.gov/dokuwiki/doku.php?id=peptidew:lib:yeast_it |
| NIST | Yeast  (S.cerevesiae) | 1, 000 | Yeast Collision Cell (QTOF) Library.  1,000 samples were used for testing. | https://chemdata.nist.gov/dokuwiki/doku.php?id=peptidew:lib:yeast_qtof |
| NIST | Yeast  (Pombe) | 10,000 | Yeast Pombe Ion Trap Library.  10,000 samples were used for testing. | https://chemdata.nist.gov/dokuwiki/doku.php?id=peptidew:lib:yeastpombe |
| ProteomeXchange | H.sapiens  E. coli extract | - | UPS2. UPS2 set includes six groups of eight human proteins spanning six orders of magnitude in concentration. | ftp://ftp.pride.ebi.ac.uk/pride/data/archive/2013/12/PXD000602 |
| HLA dataset (PXD05527)  with Target-decoy data | H.sapiens |  | The spectra correspond mainly to short (8–12 amino acids) non‑tryptic human HLA class I peptides, including common modifications (for example, carbamidomethylation and methionine oxidation) | ftp://ftp.pride.ebi.ac.uk/pride/data/archive/2024/08/PXD055277 |

**Fig A.** Plot of Precision-Recall curves for all validation datasets, demonstrated at the peptide level.


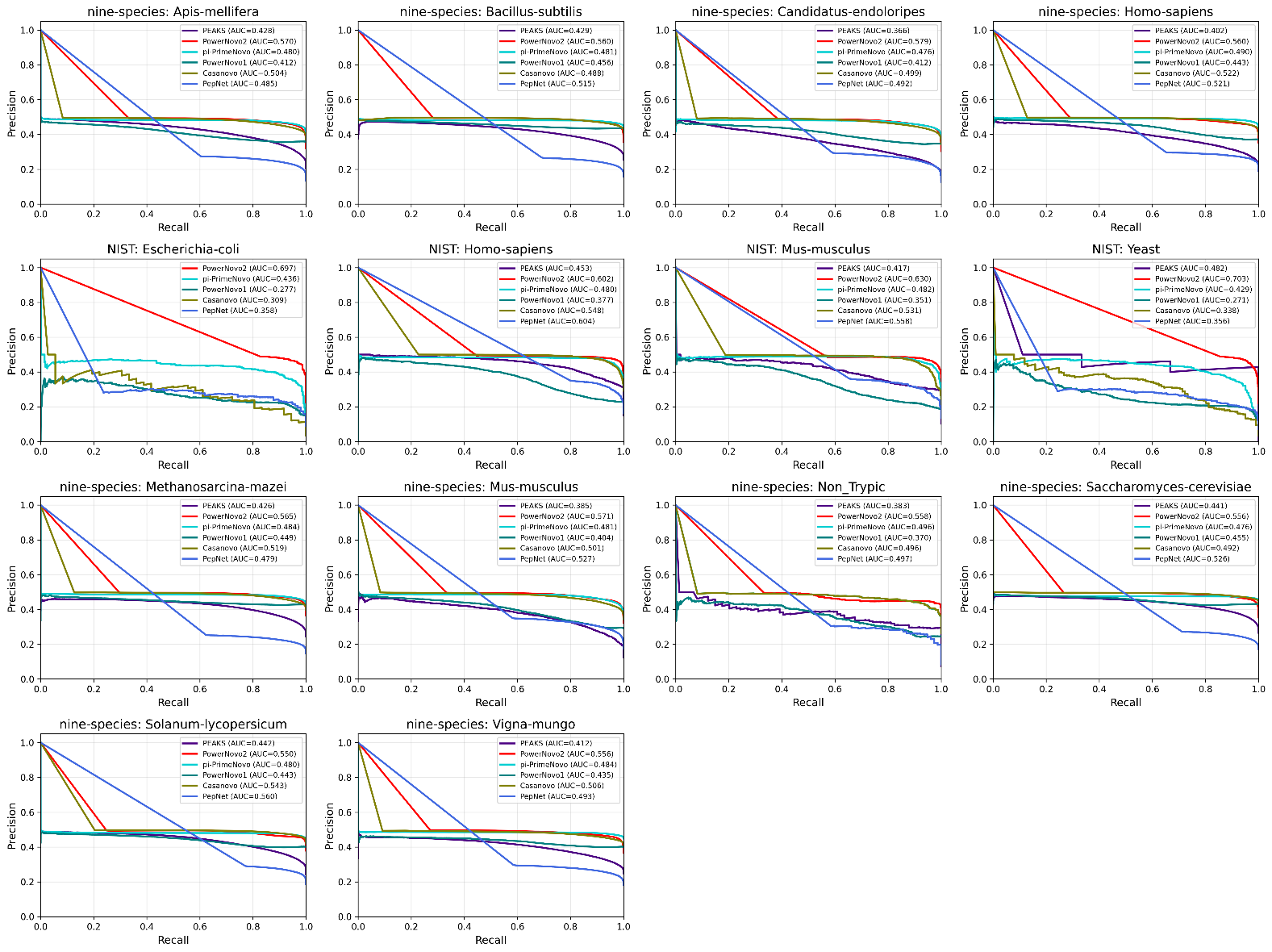


**Fig B.** Plot of Precision-Recall curves for all validation datasets, demonstrated at the amino acid level.


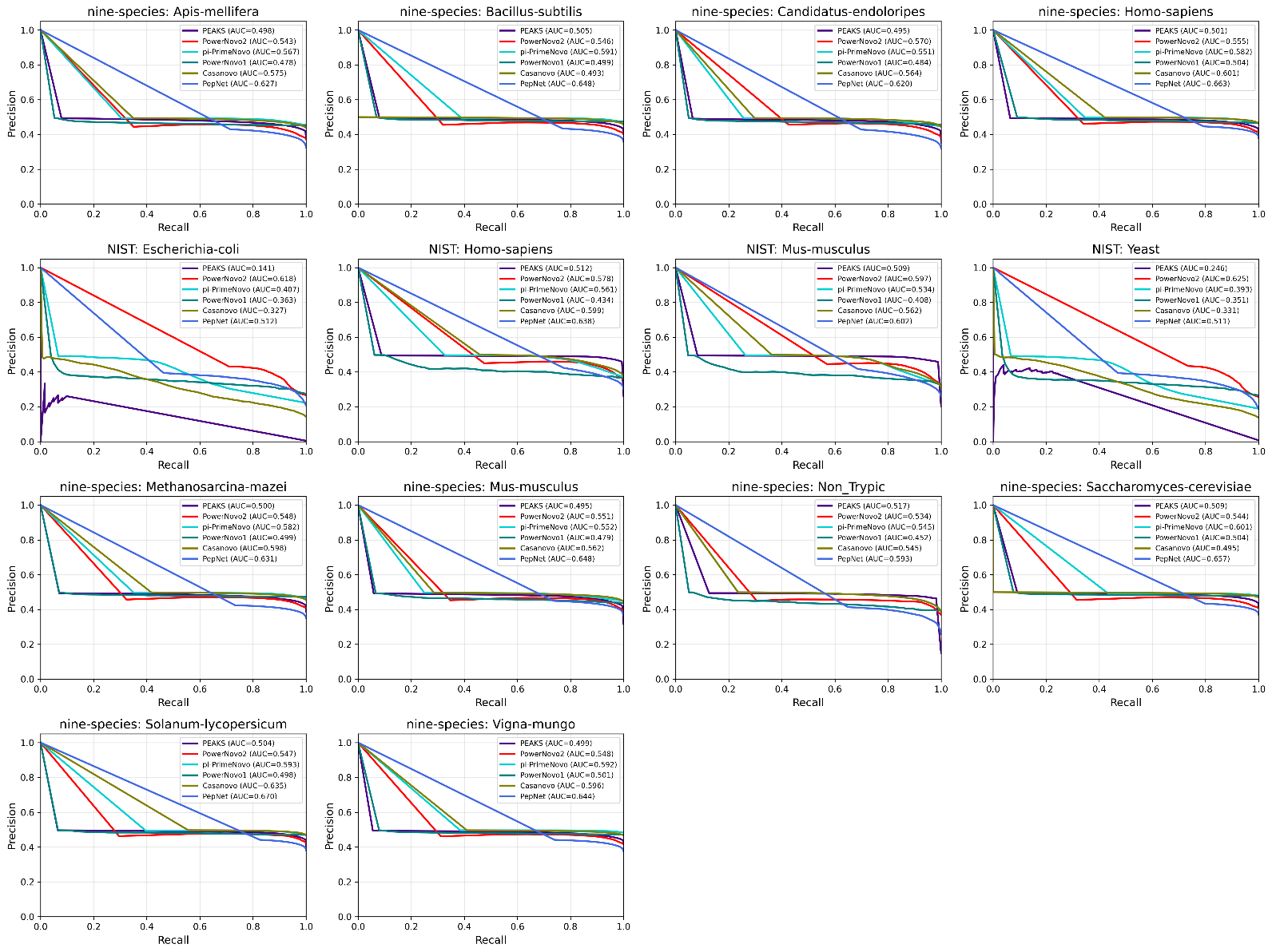


**Table C.** The percentage of protein coverage by peptides presented at various concentrations in the Escherichia coli matrix (UPS2 dataset) using *de novo* sequencing tools: Casanovo v.4.3.0, PowerNovo1, PepNet, PEAKS Studio 11, MaxQuant 1.2.2.5, π-PrimeNovo and PowerNovo2 (this study). The results are presented in t

| **Uniprot ID** | **CASANOVO** | **MAXQUANT** | **PEAKS** | **PEPNET** | **PI-PRIMENOVO** | **POWERNOVO1** | **POWERNOVO2** | **log10 mole fraction** | **Concentra**  **tion** |
| --- | --- | --- | --- | --- | --- | --- | --- | --- | --- |
| P69905ups | 0 | 92.2 | 0 | 23.4 | 15.6 | 34.75 | 17.73 | -1.9038 | 50000 |
| P68871ups | 11.64 | 94.52 | 0 | 52.74 | 41.78 | 60.96 | 29.45 | -1.9038 | 50000 |
| P41159ups | 0 | 23.13 | 0 | 6.8 | 6.8 | 6.8 | 0 | -1.9038 | 50000 |
| P62988ups | 0 | 0 | 0 | 0 | 0 | 0 | 0 | -1.9038 | 50000 |
| P02768ups | 15.75 | 59.25 | 5.31 | 45.89 | 42.64 | 28.42 | 49.14 | -1.9038 | 50000 |
| P00915ups | 3.46 | 41.15 | 0 | 15.77 | 16.54 | 19.62 | 19.62 | -1.9038 | 50000 |
| P01031ups | 9.46 | 41.89 | 0 | 17.57 | 17.57 | 9.46 | 0 | -1.9038 | 50000 |
| P00918ups | 6.56 | 40.15 | 0 | 11.2 | 13.9 | 16.22 | 23.17 | -1.9038 | 50000 |
| P01133ups | 0 | 0 | 0 | 0 | 0 | 0 | 0 | -2.9038 | 5000 |
| P63165ups | 0 | 0 | 0 | 0 | 0 | 0 | 0 | -2.9038 | 5000 |
| P62937ups | 0 | 0 | 0 | 0 | 0 | 0 | 0 | -2.9038 | 5000 |
| P04040ups | 1.71 | 30.04 | 0 | 16.16 | 11.6 | 11.6 | 10.46 | -2.9038 | 5000 |
| P00167ups | 0 | 0 | 0 | 0 | 0 | 0 | 0 | -2.9038 | 5000 |
| P02144ups | 0 | 35.95 | 0 | 0 | 0 | 0 | 0 | -2.9038 | 5000 |
| Q06830ups | 3.54 | 14.14 | 0 | 3.54 | 3.54 | 3.54 | 0 | -2.9038 | 5000 |
| P15559ups | 2.93 | 16.48 | 0 | 5.86 | 5.86 | 5.49 | 5.86 | -2.9038 | 5000 |
| P16083ups | 0 | 26.96 | 0 | 0 | 0 | 0 | 0 | -3.9038 | 500 |
| P63279ups | 0 | 15.82 | 0 | 0 | 0 | 0 | 0 | -3.9038 | 500 |
| P06732ups | 0 | 33.6 | 0 | 0 | 0 | 0 | 0 | -3.9038 | 500 |
| P12081ups | 0 | 0 | 0 | 0 | 0 | 0 | 0 | -3.9038 | 500 |
| P00709ups | 0 | 0 | 0 | 0 | 0 | 0 | 0 | -3.9038 | 500 |
| P02753ups | 0 | 34.08 | 0 | 5.59 | 0 | 0 | 0 | -3.9038 | 500 |
| Q15843ups | 0 | 0 | 0 | 0 | 0 | 0 | 0 | -3.9038 | 500 |
| P61626ups | 0 | 11.54 | 0 | 0 | 0 | 0 | 0 | -3.9038 | 500 |
| P10599ups | 0 | 0 | 0 | 0 | 0 | 0 | 0 | -4.9038 | 50 |
| P61769ups | 0 | 0 | 7.07 | 0 | 0 | 0 | 0 | -4.9038 | 50 |
| P01344ups | 0 | 0 | 0 | 0 | 10.45 | 0 | 0 | -4.9038 | 50 |
| P01127ups | 0 | 0 | 0 | 0 | 0 | 0 | 0 | -4.9038 | 50 |
| P08263ups | 0 | 0 | 0 | 0 | 0 | 0 | 0 | -4.9038 | 50 |
| P55957ups | 4.1 | 0 | 0 | 0 | 0 | 0 | 3.59 | -4.9038 | 50 |
| O76070ups | 0 | 36.05 | 0 | 0 | 0 | 0 | 0 | -4.9038 | 50 |
| P01008ups | 0 | 0 | 0 | 0 | 0 | 0 | 0 | -4.9038 | 50 |
| P01112ups | 0 | 0 | 0 | 0 | 0 | 0 | 0 | -5.9038 | 5 |
| P99999ups | 0 | 0 | 0 | 0 | 0 | 0 | 0 | -5.9038 | 5 |
| P02787ups | 0 | 0 | 0 | 0 | 0 | 0 | 0 | -5.9038 | 5 |
| P06396ups | 0 | 0 | 0 | 0 | 0 | 0 | 0 | -5.9038 | 5 |
| O00762ups | 0 | 0 | 0 | 0 | 0 | 0 | 0 | -5.9038 | 5 |
| P09211ups | 0 | 0 | 0 | 0 | 0 | 0 | 0 | -5.9038 | 5 |
| P51965ups | 0 | 0 | 0 | 0 | 0 | 0 | 0 | -5.9038 | 5 |
| P01579ups | 0 | 0 | 0 | 0 | 0 | 0 | 0 | -5.9038 | 5 |
| P02788ups | 0 | 3.47 | 0 | 0 | 0 | 0 | 0 | -6.9038 | 0.5 |
| P08758ups | 0 | 0 | 0 | 2.19 | 0 | 0 | 2.19 | -6.9038 | 0.5 |
| P02741ups | 0 | 0 | 0 | 0 | 0 | 0 | 0 | -6.9038 | 0.5 |
| P05413ups | 0 | 0 | 0 | 0 | 0 | 0 | 0 | -6.9038 | 0.5 |
| P01375ups | 0 | 0 | 0 | 0 | 0 | 0 | 0 | -6.9038 | 0.5 |
| P10636-8ups | 0 | 0 | 0 | 0 | 0 | 0 | 0 | -6.9038 | 0.5 |
| P00441ups | 0 | 0 | 0 | 0 | 0 | 0 | 0 | -6.9038 | 0.5 |

*The sensitivity of de novo sequencing models to protein peptides across a broad concentration range in biological samples was assessed using the UPS2 reference dataset (Universal proteomics standard set, Sigma-Aldrich)*[2]*.*

### ***Target–decoy dataset for the HLA data (decoy spectra)***

To assess the calibration of confidence scores and the ability of PowerNovo2 to distinguish informative spectra from random matches, we used the target–decoy strategy introduced in NovoBoard on the same HLA dataset (PXD055277). For each original (“target”) MS/MS spectrum, a corresponding decoy spectrum was generated by partially randomizing the fragment peaks: a fixed fraction of peaks (typically 50–70%) was removed from the original spectrum and replaced with “noise” peaks randomly sampled from the global pool of peaks across the dataset. This procedure preserves the overall m/z range, total peak count, and intensity distribution, while disrupting the informative fragmentation patterns required for correct peptide reconstruction. As a result, each scan has a matched target and decoy spectrum with similar precursor properties and noise characteristics, enabling construction of score distributions for true and false de novo peptide–spectrum matches and empirical FDR estimation without relying on a sequence database. In our study, we used the decoy MGF files and generation settings described by Tran et al., and applied PowerNovo2 (and π‑PrimeNovo) to both target and decoy spectra to obtain target–decoy score distributions and to estimate FDR at different levels of spectrum perturbation (50%, 60%, and 70% peak replacement).

### ***References***

1. Vaswani, A.; Shazeer, N.; Parmar, N.; Uszkoreit, J.; Jones, L.; Gomez, A.N.; Kaiser, L.; Polosukhin, I. Attention Is All You Need. *arXiv:1706.03762 [cs]* **2017**.

2. Krey, J.F.; Wilmarth, P.A.; Shin, J.-B.; Klimek, J.; Sherman, N.E.; Jeffery, E.D.; Choi, D.; David, L.L.; Barr-Gillespie, P.G. Accurate Label-Free Protein Quantitation with High- and Low-Resolution Mass Spectrometers. *J. Proteome Res.* **2014**, *13*, 1034–1044, doi:10.1021/pr401017h.
